# Radiological Findings for Distinguishing Between Xanthogranulomatous Cholecystitis and Gallbladder Cancer

**DOI:** 10.34172/aim.31710

**Published:** 2024-12-01

**Authors:** Ahmet Bozer, Nagihan Durgun

**Affiliations:** ^1^Department of Radiology, Izmir City Hospital, Izmir, Turkey

**Keywords:** Computed tomography, Differential diagnosis, Gallbladder cancer, Radiology, Xanthogranulomatous cholecystitis

## Abstract

**Background:**

Xanthogranulomatous cholecystitis (XGC) is a rare, chronic gallbladder inflammation often mistaken for gallbladder cancer (GBC) on imaging. Accurate differentiation is vital for appropriate treatment. This study aims to enhance computed tomography (CT) scan diagnostic accuracy for distinguishing XGC from GBC.

**Methods:**

This retrospective study included patients diagnosed with XGC and GBC between 2014 and 2023. CT images of 70 patients (16 GBC, 54 XGC) were reviewed. Radiologists assessed CT parameters: gallbladder wall thickening, intramural hypoattenuating nodules, enhancement characteristics, mucosal line continuity, pericholecystic fat stranding, presence of stones, bile duct dilatation, hepatic invasion, invasion to adjacent structures, and lymph node size.

**Results:**

Among 70 patients, there were 38 males (54%) and 32 females (46%), with a median age of 62 years. GBC patients were significantly older (median age 72 years) compared to XGC patients (60 years) (*P*=0.001). Diffuse gallbladder wall thickening was more frequent in XGC (70%) than GBC (12.5%) (*P*<0.001). Continuous mucosal lines and intramural hypoattenuating nodules were more common in XGC (*P*<0.001 and *P*=0.010, respectively). Intrahepatic bile duct dilatation and invasion to adjacent structures were significantly linked with GBC (*P*<0.001 and *P*=0.043). Lymph nodes with a short axis>8 mm indicated GBC (*P*<0.001), with a cutoff providing 71.4% sensitivity and 84% specificity (AUC: 0.843, *P*<0.001). CT showed 75% sensitivity (95% CI: 48-93%), 74% specificity (95% CI: 60%-85%), and 74% accuracy (95% CI: 62%-84%).

**Conclusion:**

CT imaging can effectively differentiate XGC from GBC, and larger studies can further improve diagnostic accuracy.

## Introduction

 Xanthogranulomatous cholecystitis (XGC) stands out as an uncommon type of chronic cholecystitis marked by inflammation within the gallbladder, accompanied by infiltration of both acute and chronic inflammatory cells.^1^ A defining feature of this condition is the accumulation of lipid-laden macrophages within the gallbladder wall.^2^ Consequently, XGC frequently results in substantial adhesions between the gallbladder and neighboring structures such as the duodenum, colon, and stomach. This inflammatory process can mimic gallbladder cancer (GBC), posing diagnostic challenges for both clinicians and radiologists.

 XGC is an uncommon condition, with a prevalence estimated to be between 0.7% and 10%.^3^ While GBC, which shares similar clinical and radiological features with XGC, is infrequent, based on the GLOBOCAN 2018 data, GBC represents only 1.2% of all global cancer diagnoses but accounts for 1.7% of all cancer-related fatalities. It is frequently identified at an advanced stage, with an average survival of less than one year for advanced-stage cancer.^4^

 In both of these rare diseases, patients present with similar symptoms such as pain, obstructive jaundice, cholangitis, and a palpable mass. Distinguishing between these two conditions through laboratory tests is quite challenging. Although elevated tumor markers are observed in XGC, it can often lead to concerns among surgeons due to the potential confusion with GBC.^5,6^ Therefore, the commonly detected high levels of CA19-9 in XGC may not reliably differentiate malignancy. The increase in CA19-9 can be attributed to the epithelial cell damage in the gallbladder wall and bile ducts caused by inflammation, particularly in obstructive cases. Hence, preoperative radiological findings play a crucial role in the diagnostic challenge posed by these two gallbladder pathologies.

 Studies have investigated the use of diagnostic tools such as contrast-enhanced ultrasound (CEUS), diffusion-weighted imaging (DWI) added to conventional and multiparametric magnetic resonance imaging (MRI), and positron emission tomography-computed tomography (PET/CT), alongside standard modalities like ultrasound (US), computed tomography (CT), and MRI, in the preoperative differentiation of benign and malignant gallbladder lesions.^7-9^ In one study, Lee et al^10^ found MRI to be superior to CT and US among conventional diagnostic tools in differentiating XGC from gallbladder carcinoma. Bo et al^11^ reported that CEUS demonstrated the highest diagnostic performance, followed by abdominal US, MRI, CT, and PET/CT. Similarly, Sabaté-Llobera et al^12^ found PET/CT to have comparable accuracy to conventional imaging techniques in distinguishing between benign and malignant gallbladder lesions. In light of these findings, the diagnostic performance and superiority of different modalities remain inconclusive.

 This research seeks to enhance the accuracy of CT, a pivotal radiological tool, in differentiating between XGC and GBC. By identifying and analyzing distinct imaging features, it aims to improve the accuracy of preoperative diagnoses, thereby aiding in the effective clinical management of these rare pathologies. Our research will explore the diagnostic capabilities of CT and propose specific criteria that can enhance its utility in clinical settings. The findings aim to provide radiologists and clinicians with better tools to distinguish between XGC and GBC and make informed decisions regarding patient care.

## Materials and Methods

###  Patient Selection

 Our study was designed retrospectively following approval from the ethics committee (approval number: 2023/196, dated 15.11.2023). Patients diagnosed with XGC and GBC between 2014 and 2023 were included in our study. During the specified years, 35 patients were diagnosed with GBC, while 92 patients were confirmed to have XGC.

 The exclusion criteria were as follows: absence of adequate imaging in our hospital’s CT scan protocol (27 patients), presence of only non-contrast images due to various reasons (10 patients), images being too artifact-laden for evaluation (8 patients), history of malignancy other than GBC (4 patients), inadequate medical records (7 patients), and diagnosis of both XGC and GBC (1 patient). Eventually, 16 patients remained in the GBC group, while 54 patients remained in the XGC group.

###  Imaging Technique

 Dynamic imaging was performed on patients included in our study using the Siemens SOMATOM Definition AS 64 CT scanner. The protocol consisted of 128 slices with a collimation of 0.625 and a slice thickness of 5 mm. The pitch was set to 0.9 with a rotation time of 0.5 seconds at 120 kVp and 150-200 mAs.

 A non-ionic contrast agent (Ultravist 370 mg iodine/mL, Berlin, Germany) was administered at a rate of 1-2 mL/kg using an automatic injector at a speed of 3-4 mL/s. Imaging was conducted at 30 seconds post-contrast for the arterial phase and 60 seconds post-contrast for the portovenous phase.

###  Imaging Interpretation

 All images were evaluated by two radiologists with 9 and 4 years of experience in abdominal radiology, who reached a consensus. In cases where a decision could not be reached, our abdominal radiologist with 30 years of experience provided guidance.

**Figure 1:**
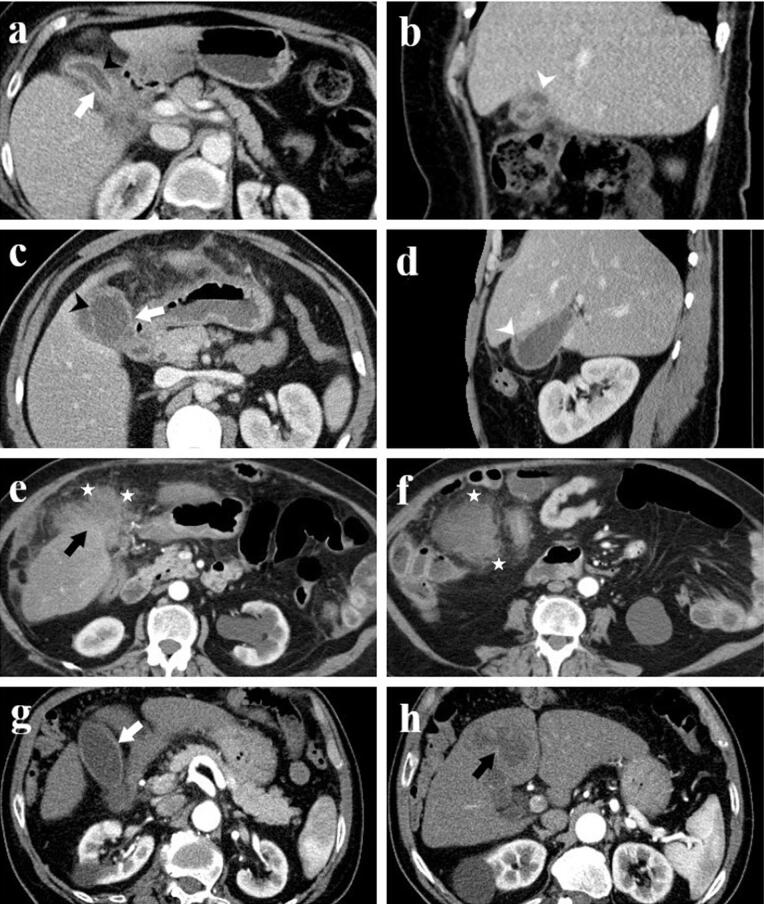
Computed Tomography Images of Four Patients with Histopathologically Confirmed Xanthogranulomatous Cholecystitis. Axial (a) and sagittal (b) images from patient 1; axial (c) and sagittal (d) images from patient 2; axial images (e, f) from patient 3; and axial images (g, h) from patient 4. The images illustrate findings such as diffuse gallbladder wall thickening (white arrow), continuity of the mucosal line (black arrowhead), intramural hypodense nodules (white arrowhead), pericholecystic infiltration (asterisk), and an indistinct interface suggesting infiltration into the liver parenchyma (black arrow)

**Figure 2:**
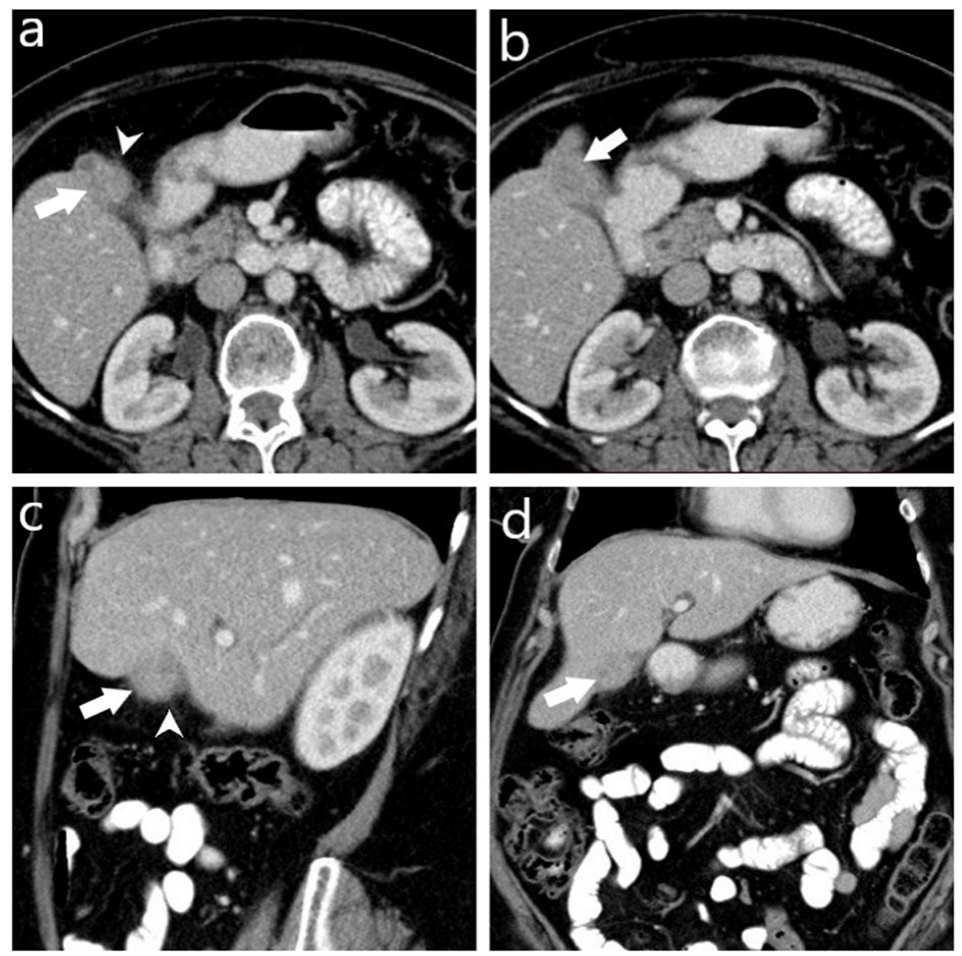
Computed Tomography Images of a Histopathologically Diagnosed Case of Xanthogranulomatous Cholecystitis Preoperatively Misdiagnosed as Gallbladder Carcinoma. Axial (a, b), sagittal (c), and coronal (d) post-contrast portal venous phase CT images show a collapsed gallbladder with asymmetrical wall thickening and a mass-like appearance (arrow), and minimal pericholecystic fat stranding (arrowhead). No regional lymph node enlargement or invasion to the liver and adjacent structures is observed. However, characteristic features suggestive of XGC, such as intramural hypodense nodules and continuity of the mucosal line, are not present

**Figure 3:**
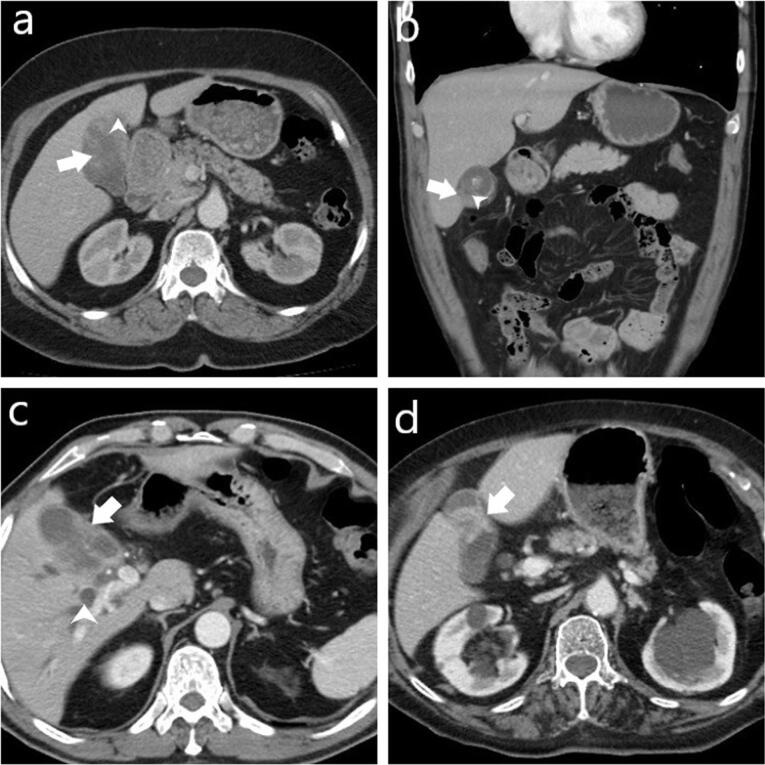
Computed Tomography Images of Four Cases Histopathologically Diagnosed with Gallbladder Carcinoma. (a) Shows a mass lesion (arrow) originating from the gallbladder wall with polypoid extension into the lumen and invasion into the adjacent liver parenchyma (arrowhead). (b) Demonstrates focal thickening of the gallbladder (arrow) and disrupted mucosal line (arrowhead). (c) Displays asymmetrical wall thickening (arrow) and intrahepatic bile duct dilatation (arrowhead). (d) Reveals focal concentric thickening of the gallbladder wall (arrow)

 The following parameters were evaluated in the patients’ images:

- Presence of gallbladder wall thickening, including thickness (mm), and wall thickness pattern (focal, diffuse, polypoid, massive),
- Assessment of the presence of intramural hypoattenuating nodules in the thickened wall,
- Evaluation of enhancement characteristics of the gallbladder wall (homogeneous or heterogeneous),
- Examination of continuity of mucosal lines (continuous or disrupted),
- Presence of pericholecystic fat stranding and infiltration,
- Assessment of stones in the gallbladder and bile ducts,
- Examination of intra-extrahepatic bile duct dilatation,
- Assessment of macroscopic hepatic invasion and extent,
- Assessment of invasion to adjacent structures (duodenum, hepatic flexure of the colon),
- Finally, the presence of regional lymph nodes was assessed. If present, the short-axis dimension of the lymph node was measured. Additionally, a separate assessment was conducted to identify lymph nodes with a short-axis dimension greater than 10 mm (Figures 1-3).

###  Statistical Analysis 

 Analysis of the data obtained in the study was conducted using IBM SPSS Statistics 26 (IBM Corp., Armonk, NY, USA). The suitability of continuous variables for normal distribution was assessed through graphical evaluation, normality tests, and sample size analysis, showing that they did not meet the conditions of normal distribution. Continuous variables were presented as median and interquartile range (IQR) (25-75). The Mann-Whitney U test was used for comparisons between independent groups. ROC analysis was performed to determine the diagnostic power of the parameters, and the optimal cut-off value was identified using Youden’s J index. Categorical variables were presented as frequencies and percentages in cross-tables, with comparisons conducted using the chi-square and Fisher’s Exact tests. For dependent categorical variables, the McNemar test was applied, and predictive values were calculated. All statistical tests were two-tailed with a type one error margin set at α: 0.05.

## Results

 Our study included 70 patients, of whom 38 (54%) were male and 32 (46%) female. The median age was 62 years (IQR: 51-72).

 In the GBC group, the median age (IQR) was found to be 72 years (64-78), while in the XGC group, it was 60 (49-68). The GBC group had a higher average age, and the difference in age between the two groups was statistically significant (*P* = 0.001) (Table 1).

**Table 1:** Comparative Patient Demographics and Radiological Features of Gallbladder Carcinoma and Xanthogranulomatous Cholecystitis

**Features**		**Total** **N (%)**	**Pathological Diagnosis**		***P*** ** Value **
			**GBC**	**XGC**	
			**N (%)**	**N (%)**	
Gender	Male	38 (54%)	7 (44%)	31 (57%)	0.498*
	Female	32 (46%)	9 (56%)	23 (43%)	
Age, Med.(IQR)		62 (51-72)	72 (64-78)	60 (49-68)	0.001**
Wall thickness (mm), Med. (IQR)		5 (4-7)	7 (3-9)	5 (4-6)	0.428**
Gallbladder wall thickening pattern	Focal	24 (34%)	8 (50%)	16 (30%)	N/A
	Diffuse	40 (57%)	2 (12.5%)	38 (70%)	
	Polypoid	4 (6%)	4 (25%)	0 (0%)	
	Massive	2 (3%)	2 (12.5%)	0 (0%)	
Focal gallbladder wall thickening	Present	24 (34%)	8 (50%)	16 (30%)	0.227
	Absent	46 (66%)	8 (50%)	38 (70%)	
Diffuse gallbladder wall thickening	Present	40 (57%)	2 (12.5%)	38 (70%)	< 0.001*
	Absent	30 (43%)	14 (87.5%)	16 (30%)	
Enhancement characteristics of gallbladder wall	Homogeneous	43 (61%)	9 (56%)	34 (63%)	0.848*
	Heterogeneous	27 (39%)	7 (44%)	20 (37%)	
Continuity of mucosal line	Continuous	44 (63%)	1 (6%)	43 (80%)	< 0.001*
	Disrupted	26 (37%)	15 (94%)	11 (20%)	
Pericholecystic fat stranding	Present	56 (80%)	14 (87.5%)	42 (78%)	0.497*
	Absent	14 (20%)	2 (12.5%)	12 (22%)	
Pericholecystic infiltratio	Present	41 (59%)	8 (50%)	33 (61%)	0.615*
	Absent	29 (41%)	8 (50%)	21 (39%)	
Intramural hypoattenuating nodules in the thickened wall	Present	35 (50%)	3 (19%)	32 (59%)	0.010*
	Absent	35 (50%)	13 (81%)	22 (41%)	
Stones (gallbladder and bile ducts)	Present	42 (60%)	10 (63%)	32 (59%)	1.000*
	Absent	28 (40%)	6 (37%)	22 (41%)	
Intrahepatic bile duct dilatation	Present	18 (26%)	12 (75%)	6 (11%)	< 0.001*
	Absent	52 (74%)	4 (25%)	48 (89%)	
Extra-hepatic bile duct dilatation	Present	16 (23%)	6 (37%)	10 (19%)	0.172*
	Absent	54 (77%)	10 (63%)	44 (81%)	
Macroscopic hepatic invasion	Present	16 (23%)	6 (37%)	10 (19%)	0.172*
	Absent	54 (77%)	10 (63%)	44 (81%)	
Macroscopic hepatic invasion extent	Indistinct border	11 (16%)	4 (25%)	7 (13%)	N/A
	Massive	5 (7%)	2 (12.5%)	3 (6%)	
	None	54 (77%)	10 (62.5%)	44 (81%)	
Invasion to adjacent structures (duodenum, hepatic flexure of the colon)	Present	7 (10%)	4 (25%)	3 (6%)	0.043*
	Absent	63 (90%)	12 (75%)	51 (94%)	
LN (regardless of size)	Present	32 (46%)	7 (44%)	25 (46%)	1.000*
	Absent	38 (54%)	9 (56%)	29 (54%)	
LN short axis dimension (mm), Med.(IQR)		7 (5-9)	11 (7-11)	6 (5-7)	0.006**
LN short axis dimension (cut-off value)	> 8 mm	9(28%)	5(71%)	4(16%)	0.010*
	≤ 8 mm	23(72%)	2(29%)	21(84%)	
LN (short axis > 10 mm)	Present	6 (9%)	4 (25%)	2 (4%)	0.022*
	Absent	64 (91%)	12 (75%)	52 (96%)	

Med: Median, IQR: Interquantile range (25-75), N/A: Not available, LN: Lymph node; GBC: Gallbladder carcinoma; XGC: Xanthogranulomatous cholecystitis. ^*^Pearson chi-square test or Fisher’s exact test, ^**^Mann-Whitney U test.

 The distribution of gallbladder wall thickening patterns among the groups is presented in Table 1. Diffuse gallbladder wall thickening was observed in 2 patients (12.5%) in the GBC group and in 38 patients (70%) in the XGC group. This difference was found to be statistically significant (*P* < 0.001). The presence of focal gallbladder wall thickening was not significantly different between the two groups (*P* = 0.227).

 A continuous mucosal line was observed in 1 patient (6%) in the GBC group and in 43 patients (80%) in the XGC group. This difference was statistically significant (*P* < 0.001).

 Additionally, intramural hypoattenuating nodules in the thickened wall were observed in 3 patients (19%) in the GBC group and in 32 patients (59%) in the XGC group, with this difference being statistically significant (*P* = 0.010).

 Among the parameters examined on CT, intrahepatic bile duct dilatation was present in 75% (n:12) of GBC patients and in 11% (n:6) of XGC patients. This difference between the two groups was statistically significant (*P* < 0.001).

 Invasion to adjacent structures (duodenum, hepatic flexure of the colon) was present in 25% (n:4) of GBC patients and in 6% (n:3) of XGC patients. This parameter was found to be statistically significant between the two groups (*P* = 0.043, Table 1).

 The median (IQR) of lymph node short axis dimension was found to be 11 (7-11) in the GBC group and 6 (5-7) in the XGC group, with statistical significance (*P* = 0.006) (Table 1).

**Figure 4:**
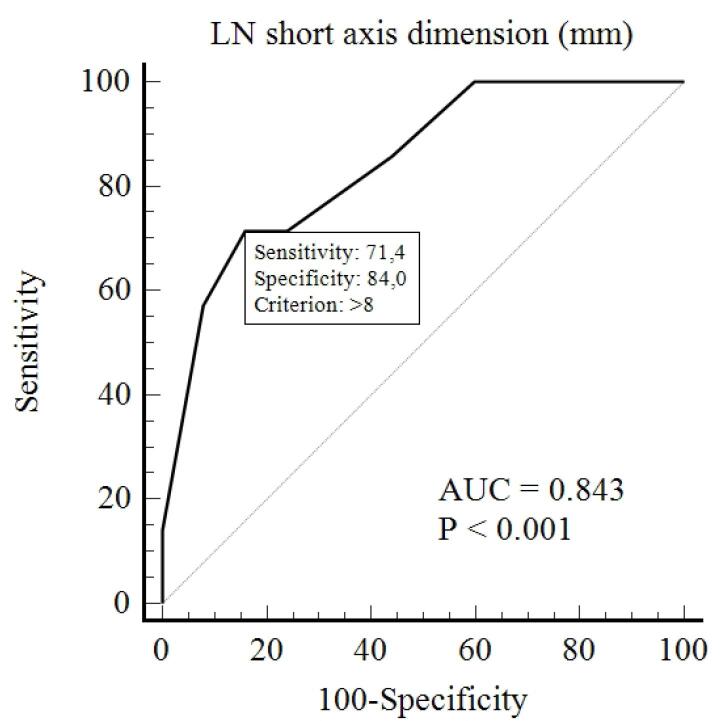
ROC Analysis for Determining the Cut-off Value for the Short Axis Dimension of Lymph Nodes

 To determine a cut-off value for the short axis of lymph nodes that can distinguish between the two groups, ROC analysis was conducted. A cut-off value of 8 mm was found with 71.4% sensitivity, 84% specificity, and an AUC of 0.843 (*P*< 0.001) (Figure 4). At this cut-off value, the distribution of lymph nodes in both groups is shown in Table 1 (*P* = 0.010).

 Other parameters examined on CT showed no statistically significant distinctions between the two groups (Table 1).

**Figure 5:**
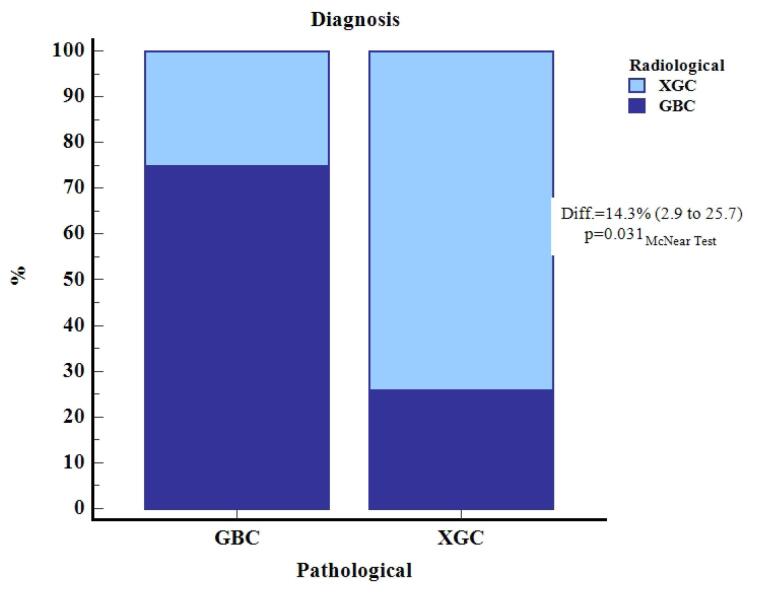
Schematic Comparison of Radiological and Pathological Diagnoses for Gallbladder Carcinoma (GBC) and Xanthogranulomatous Cholecystitis (XGC)

 The diagnostic performance of CT in distinguishing between GBC and XGC groups showed a sensitivity of 75% (95% CI: 48%-93%), a specificity of 74% (95% CI: 60%-85%), and an accuracy of 74% (95% CI: 62%-84%) (Table 2, Figure 5).

**Table 2:** Diagnostic Performance of Computed Tomography for Gallbladder Carcinoma and Xanthogranulomatous Cholecystitis

	**Value**	**95% CI**
Sensitivity	75%	48% to 93%
Specificity	74%	60% to 85%
AUC	0.75	0.63 to 0.84
Positive Likelihood Ratio	2.89	1.70 to 4.93
Negative Likelihood Ratio	0.34	0.14 to 0.80
Positive Predictive Value	46%	33% to 59%
Negative Predictive Value	91%	81% to 96%
Accuracy	74%	62% to 84%

 The concordance between radiological and pathological diagnoses of GBC and XGC reveals that out of the 70 total cases, radiological diagnosis correctly identified 12 cases of GBC (17.1%) and 40 cases of XGC (57.1%). However, it misclassified 4 cases of GBC as XGC (5.7%) and 14 cases of XGC as GBC (20.0%) (Table 3). The McNemar test indicated a statistically significant difference (*P* = 0.031), with a difference in diagnostic accuracy of 14.3% (95% CI: 2.9% to 25.7%) (Table 3).

**Table 3:** Concordance between Radiological and Pathological Diagnoses of Gallbladder Carcinoma and Xanthogranulomatous Cholecystitis

		**Pathological Diagnosis**				**Total **	
		**GBC**		**XGC**			
		**N**	**(Tot.) %**	**N**	**(Tot.) %**	**N**	**(Tot.) %**
Radiological diagnosis	GBC	12	17.1	14	20.0	26	37.1
	XGC	4	5.7	40	57.1	44	62.9
Total		16	22.9	54	77.1	70	100.0

GBC: Gallbladder carcinoma; XGC: Xanthogranulomatous cholecystitis. McNemar test *P* value: 0.031_, _Difference (95% CI): 14.3% (2.9 to 25.7).

## Discussion

 Preoperative differentiation between XGC and GBC based on clinical, laboratory, and radiological findings can be challenging. However, it is vital to make this distinction. Misdiagnosis can result in inappropriate surgical interventions, like opting for a radical cholecystectomy and organ resection for XGC or a simple laparoscopic cholecystectomy for suspected malignancies. Although ultrasound-guided fine-needle aspiration (FNA) has been attempted preoperatively, a negative FNA does not exclude GBC, if the sampled area does not represent the lesion.^13,14^ Some studies have recommended ultrasound-guided FNA in cases of focal or diffuse gallbladder wall thickening. However, due to operator dependency and the associated risks of tumor dissemination and fistula formation, it is not used routinely.^15^

 Intraoperative frozen section is regarded as the gold standard for distinguishing between these two conditions, despite concerns regarding time and cost.^16,17^ However, due to the resemblance between XGC and GBC, radical surgery, including organ resection, might still be performed in XGC cases, leading to high morbidity and mortality. Therefore, while preoperative radiological differentiation between XGC and GBC is important, it remains challenging. Accurate radiological assessment can help guide the surgical approach, potentially avoiding inappropriate surgery and improving patient management, though limitations in imaging must be acknowledged.

 GBC typically manifests after the age of 60 due to the prolonged development of malignancy over many years.^18^ Similarly, XGC tends to occur in older adults.^3^ However, in our study, the median age of the GBC group was higher than that of the XGC group, and the age distribution between the two groups was found to be statistically significant. In the literature, some studies have found no statistically significant difference in age between these two groups.^16,19,20^ However, Rajaguru et al, in their scoring system developed to distinguish between the two groups, found that age above 55 was a significant factor for GBC, while an age below 55 was significant for XGC.^21^

 Diffuse gallbladder wall thickening, continuity of the mucosal line, and intramural hypoattenuating nodules in the thickened wall were found to be significant for XGC. There is sufficient literature suggesting that these radiological features are diagnostic for XGC.^16,22-24^ However, as observed in our study, these features can also be occasionally present in the GBC group.

 In our study, absence of intrahepatic bile duct dilatation (*P* < 0.001), absence of invasion to adjacent structures (duodenum, hepatic flexure of the colon) (*P* = 0.043), and short axis of regional lymph nodes less than 8 mm (*P* = 0.010) were also found to be significant for XGC. However, there are studies in the literature indicating that intrahepatic bile duct dilatation is not significant in distinguishing between the two groups.^2,16^ In XGC cases, severe inflammation and adhesions can lead to bile duct dilatation due to compressive effects in the chronic phase.

 In our study, macroscopic hepatic invasion was observed in 6 patients (37%) in the GBC group and 10 patients (19%) in the XGC group, with no significant difference between the groups (*P* = 0.172). Although the absence of macroscopic hepatic invasion suggests XGC, pseudo-tumoral presentations of XGC can exhibit extension to adjacent organs and structures.^23,25^ Additionally, invasion to adjacent structures (duodenum, hepatic flexure of the colon) was found to be statistically significant between the two groups.

 The median measurement of the short axis of regional lymph nodes was found to be statistically significant between the two groups. Subsequently, a cutoff value of 8 mm for the short axis of regional lymph nodes was calculated to distinguish between the two groups. This means that a short axis measurement of regional lymph nodes above 8 mm suggests GBC, while measurements equal to or below 8 mm suggest XGC (71.4% sensitivity, 84% specificity). Additionally, our study found that lymph nodes with a short axis greater than 10 mm were statistically significant between the two groups (*P* = 0.022) (Table 1).

 Consistent with our study, some previous research has demonstrated that enlarged lymph nodes can be used to distinguish between the two groups.^16,26^ In a cohort study comprising 60 cases, Wasnik et al^26^ identified a correlation between lymph node size and the likelihood of malignancy when distinguishing GBC from acute cholecystitis and XGC. However, there are also studies in the literature that have found enlarged lymph nodes in the XGC group, concluding that they cannot be used to differentiate from GBC.^22,27^

 GBC lymph node metastasis is one of the most common types of metastasis and is the most important factor affecting the clinical staging of GBC. A lymph node with a diameter greater than 1 cm detected by imaging methods is considered a positive criterion for lymph node metastasis.^28^ The 1-cm value here is used for metastatic involvement. However, the cut-off value we found in our study, which is 8 mm, can be used with high sensitivity and specificity to differentiate between the XGC and GBC groups. Except for our study, there is no research in the literature that determines the cutoff value for lymph node short-axis measurement to differentiate between XGC and GBC.

 In our study, stones (gallbladder and bile ducts) were present in 63% (10 out of 16) of GBC cases and 59% (32 out of 54) of XGC cases. The presence of stones in the gallbladder and bile ducts was not found to be statistically significant in differentiating between the two groups. Cholelithiasis, due to chronic irritation of the mucosa, leads to mucosal metaplasia, dysplasia, and subsequently carcinoma, and is a well-known risk factor for GBC.^29^ Approximately 95% of GBCs are associated with gallstones.^30^ Similarly, the most significant association with XGC is gallstones, observed in approximately 80% of cases.^3^ These findings suggest that using the presence of gallstones to differentiate between the two groups may not be appropriate. However, some studies have found a higher prevalence of gallbladder stones in the XGC group compared to GBC, with statistical significance.^16,22,26^

 In our study, CT demonstrated reasonable diagnostic performance in distinguishing between GBC and XGC, with a sensitivity of 75%, specificity of 74%, and accuracy of 74% (Table 2). However, 14 out of 54 XGC patients were falsely reported as GBC on CT, and 4 out of 16 GBC patients were interpreted as false negatives on CT (Table 3). Similar findings have been reported in previous studies.^23,26^ This underscores the diagnostic challenge and variability in distinguishing between GBC and XGC using CT alone, as shown in Table 3.

 In a study involving 88 patients, Lee et al^10^ compared the diagnostic performance of high-resolution ultrasound (HRUS), CT, and MRI in differentiating between XGC and GBC. Despite MRI demonstrating superior performance to both HRUS and CT, HRUS exhibited better performance than CT.

 In another study, Bo et al^11^ found that CEUS exhibited superior diagnostic performance, with a sensitivity and specificity of 90% and 93%, respectively, compared to other imaging modalities including abdominal US, CT, MRI, and PET/CT, in distinguishing between XGC and GBC. They reported sensitivity and specificity values of 71% and 92%, respectively, for CT.

 Kalage et al^8^ conducted a study to investigate the diagnostic performance of a multiparametric MRI protocol for characterizing gallbladder wall thickening. They utilized a combination of DWI, intravoxel incoherent motion, diffusion tensor imaging, and dynamic contrast-enhanced MRI (DCE-MRI). Their findings demonstrated that the multiparametric protocol significantly enhanced the sensitivity (90%) and specificity (88%) for distinguishing malignant gallbladder wall thickening from benign cases, with quantitative parameters such as time to peak enhancement and mean diffusivity showing strong associations with malignancy.

 Han et al^31^ developed an MRI scoring system to differentiate XGC from GBC, achieving excellent diagnostic performance with an AUC of 0.972. This MRI scoring system incorporated nine features: diffuse gallbladder wall thickening, mucosal uniformity, intramural T2-high signal intensity, mucosal retraction, gallbladder stones, T1-intermediate to high-signal intensity, diffusion restriction, enhancement pattern, and phase of peak enhancement. Although there are studies supporting the diagnosis of XGC through the identification of intramural foamy histiocyte accumulation and fat content using the chemical shift artifact, which shows higher signal intensity on in-phase images compared to opposed-phase images,^32,33^ Han et al^31^ observed this phenomenon in only five patients (8%) in their XGC cohort. Therefore, they concluded that it should not be considered a reliable indicator of intramural fat content.

 In contrast, Ito et al^19^ developed a CT scoring system based on five CT features: diffuse wall thickening, absence of polypoid lesions, intramural nodules or bands, pericholecystic infiltration, and pericholecystic abscess. By applying three or more of these features, they achieved a sensitivity of 77% (95% CI: 57%-87%) and a specificity of 94% (95% CI 86%-98%) in distinguishing XGC from GBC. The AUC of the MRI scoring system developed by Han et al^31^ was slightly higher than that of the CT scoring system proposed by Ito et al.^19^ In our study, we identified CT features such as absence of intrahepatic bile duct dilatation, lack of invasion to adjacent structures, and smaller regional lymph nodes (less than 8 mm), which could be incorporated into the existing scoring systems to further improve diagnostic accuracy in differentiating the two groups.

 In light of these findings, our study underscores the significance of refining diagnostic approaches by integrating additional radiological features to enhance accuracy. Incorporation of the CT features identified in our study could contribute to more accurate preoperative differentiation between XGC and GBC, thereby supporting improved clinical decision-making and ultimately leading to better patient outcomes. This approach can guide more tailored treatment strategies, minimize unnecessary procedures, and optimize patient care.

 Variations in patient characteristics, including age, ethnicity, and regional healthcare access, may influence diagnostic patterns and treatment outcomes. Future multicenter studies, incorporating diverse populations from different geographic regions, would enhance the generalizability of our findings, providing a more comprehensive understanding of the clinical implications across varied settings and improving the external validity of our conclusions.

 One of the study’s limitations is its retrospective design, which could introduce selection bias and constrain the generalizability of the results. Additionally, the relatively small sample size, particularly in the GBC group, may affect the statistical power of the analysis. Lastly, the single-center nature of the study may limit the external validity of the results and necessitate further validation in larger, multicenter cohorts to confirm the generalizability of the identified radiological findings. The patient population in this study had a relatively high average age, which, along with factors such as motion artifacts, may have limited the acquisition of optimal imaging quality. Additionally, comorbidities and contraindications, such as impaired renal function limiting the use of contrast agents, led to further exclusions from the study. A strict exclusion criterion was applied to ensure the homogeneity of the study population and minimize the potential impact of these factors on the results.

## Conclusion

 In conclusion, differentiation between XGC and GBC remains a challenging task due to their overlapping clinical and radiological features. Our findings highlight that specific radiological parameters, such as diffuse gallbladder wall thickening, the continuity of the mucosal line, and the presence of intramural hypoattenuating nodules, are significantly associated with XGC. Furthermore, absence of intrahepatic bile duct dilatation, lack of invasion to adjacent structures, and smaller regional lymph nodes (less than 8 mm) further distinguish XGC from GBC. Despite the reasonable diagnostic performance of CT, with sensitivity and specificity around 75%, the study also highlights the potential for misclassification. These findings emphasize the importance of integrating comprehensive radiological assessments to improve preoperative differentiation, thereby guiding appropriate surgical interventions and enhancing patient outcomes. Further research with larger sample sizes and multi-center collaboration is essential to enhance diagnostic accuracy and develop more robust criteria for differentiating XGC from GBC.
